# Associations between small-area sociodemographic characteristics and intimate partner violence in Montréal, Québec

**DOI:** 10.1177/22799036231208326

**Published:** 2023-11-06

**Authors:** Paul Rodrigues, Mylène Fernet, Marie-Marthe Cousineau, Mathieu Philibert

**Affiliations:** 1 Institut Santé et Société, Université du Québec à Montréal, Montréal, Québec, Canada; 2 Département de Sexologie, Université du Québec à Montréal, Montréal, Québec, Canada; 3 École de Criminologie, Université de Montréal, Montréal, Québec, Canada

**Keywords:** Intimate partner violence, neighbourhoods, social inequalities, social disorganization theory, domestic violence

## Abstract

**Background:**

Intimate partner violence (IPV) affects many individuals and can have a significant impact on their health and well-being. In order to inform prevention strategies, several studies have focused on the determinants of IPV. However, knowledge on the association between neighbourhood characteristics and IPV remains scarce. The social disorganization theory posits that certain neighbourhood characteristics are associated with violent behaviours. This theory has been used to explain spatial variations in IPV, but most studies have been conducted in the United States. Little is known about the effect of neighbourhood factors in urban contexts outside of the United States.

**Design and methods:**

Using police data from 2016 and 2017, this study estimated the association between sociodemographic characteristics of neighbourhoods (socioeconomic status, single-parenthood, residential instability and ethnocultural heterogeneity) and IPV victimization in Montréal, Québec.

**Results:**

Results suggest a neighbourhood-level variation in IPV, and that neighbourhood-level characteristics are associated with IPV victimization. Specifically, the likelihood of IPV is higher in neighbourhoods with the lowest SES level (OR = 2.80, 95%CI: 2.47–3.17, *p* < 0.001) and the lowest level of residential instability (OR = 0.81, 95%CI: 0.70–0.93, *p* = 0.003) as well as the highest proportion of single-parent households (OR = 1.88, 95%CI: 1.65–2.15, *p* < 0.001).

**Conclusion:**

Although neighbourhood-level interventions to reduce IPV are rare, our results highlight the importance of developing such preventive strategies. Prevention programs targeting high-risk neighbourhoods may prove effective in reducing IPV.

## Significance for public health

Intimate partner violence affects millions of people every year. It has a significant impact on individuals (e.g. anxiety, depression, post-traumatic stress symptoms) and generates many costs for society (e.g. medical services, work productivity). Studies on the determinants of intimate partner violence have mainly focused on individual and family factors, and research on neighbourhood factors is still limited. This study highlights the role of neighbourhood sociodemographic characteristics on intimate partner violence in Montréal, Québec. Results show that IPV is higher in neighbourhoods characterized by a low SES and a high proportion of single-parent households, suggesting the importance of targeting these risky neighbourhoods in order to reduce the prevalence of this phenomenon.

## Introduction

Intimate partner violence (IPV) is a major social and public health concern around the world.^1^ IPV is a form of domestic violence characterized by psychological, physical or sexual abuse on either a current or former intimate partner.^2^ IPV has significant consequences on the health and well-being of victims. IPV victims are more likely to suffer from mental disorders such as post-traumatic stress, anxiety and depression as well as to adopt risky behaviours such as alcohol or drug abuse.^3,4^ They are also more likely to report physical injuries and chronic pain.^4^

IPV is a widespread phenomenon disproportionally affecting women.^1^ In the United States, one out of four women and one out of ten men experienced at least one form of violence in their lifetime and reported negative impacts on their health and well-being.^5^ In the province of Québec, Canada, women represent 8 out of 10 victims of offences against the person occurring in an intimate context and reported to the police.^6^ Based on the Canadian General Social Survey, 3.5% of individuals aged 15 years and over in the province of Québec were victims of physical or sexual violence at the hands of an intimate partner in the past 5 years.^7^

In comparison to individual and family factors, research on neighbourhood-level determinants of IPV is recent. However, a growing number of studies, mostly conducted in the U.S, have analysed neighbourhood-level factors in relation to IPV, and recent literature reviews suggest that different neighbourhood characteristics are associated with IPV.^8–11^ In these studies, the influence of local environments (i.e., neighbourhoods) on IPV the social disorganization theory (SDT)^12^ is the conceptual framework most frequently referred to.^8,9^ This theory posits that neighbourhood sociodemographic characteristics, specifically socioeconomic status (SES), single parenthood, residential instability and ethnocultural heterogeneity, are associated with crime and violence.^12^ However, with regard to IPV, most studies focused on the influence of neighbourhood SES, and knowledge about the effect of other neighbourhood sociodemographic characteristics (i.e. single parenthood, residential instability and ethno-cultural heterogeneity) is still limited.^10^

### Social disorganization theory and intimate partner violence

According to the SDT, SES as well as levels of single parenthood, residential instability and ethnocultural heterogeneity in neighbourhoods could influence violence, including IPV, through several psychosocial processes. The effect of neighbourhood SES on violence would be partly mediated by stress and dissatisfaction within the community as well as by access to community or public resources protecting against crime and violence.^13^ Residential instability and ethnocultural heterogeneity are said to be obstacles to the development of social ties at the local level, thereby reducing the local community’s collective efficacy (i.e., the ability of a local population to act together with a common goal such as the regulation of crime and violence in the neighbourhood).^13,14^ In effect, constant residential turnover limits the possibilities for residents to become familiar with their neighbours, while ethnocultural diversity affects the ability of residents to share common values and communicate with each other, thereby reducing social interactions between neighbours.^12^ Let us note, however, that the causal role of ethnocultural heterogeneity on the development of social ties has been a topic of debate as it has been argued that it is confounded by SES.^15,16^ Lastly, the SDT posits that neighbourhoods’ proportion of single-parent households is inversely associated with local social ties due to single parents’ lack of availability for developing and maintaining social relationships in the community, leading to lower levels of formal and informal social control.^17,18^ Social control and social ties would thus mediate the effect of neighbourhoods’ proportion of single-parent households on IPV.^13,19^

While multiple studies have examined neighbourhood-level factors in relation to crime or violence, few of them have addressed IPV specifically. Empirical studies of neighbourhood-level influences on IPV based on the SDT are few and have reported mixed results.^9^ In accordance with the SDT, many of these studies reported negative associations between neighbourhood-level SES and IPV.^13,20–28^ In contrast, empirical evidence for the presumed effect of residential instability on IPV is scarce.^9,10^ Some studies reported nonsignificant associations^13,28–30^ while others observed negative associations, in contradiction with the SDT.^20,23–25^ The role of ethnocultural heterogeneity is also equivocal.^9,10^ Most of the studies addressing this characteristic used the proportion of immigrants for operationalizing ethnocultural heterogeneity^13,22,24,25,28^ rather than measuring heterogeneity *per se* (two neighbourhoods with the same proportion of immigrants may present different levels of ethnocultural heterogeneity). Some of these studies reported nonsignificant associations,^13,24,25^ while others observed a negative association between the proportion of immigrants and IPV.^22,28^ Among the few studies that actually measured heterogeneity, none reported a significant association with IPV.^30^ Finally, single parenthood seems to have also been infrequently analysed in relation to IPV. One study observed a positive association between the neighbourhood-level proportion of female-headed, single-parent households and the risk of IPV for women.^17^ One obstacle to better understanding the role of single parenthood is that this factor is occasionally included in composite measures of deprivation,^23,24,26^ making it more difficult to distinguish between the independent effects of single parenthood and SES.

This study aims at estimating the association between IPV and sociodemographic features of neighbourhoods in Montréal, Québec. In accordance with the SDT, we hypothesized that the risk of IPV is negatively associated with neighbourhood-level SES, and positively associated with the neighbourhood-level proportion of single-parent households, residential instability and ethnocultural heterogeneity.

## Design and methods

### Data

Two data sources were combined for estimating the risk of IPV at the local level: data on the frequencies of IPV events and population sizes. Data on victim frequencies, which included all IPV victims aged 20 years or older, were provided by the Montreal City Police Department (SPVM, French acronym for *Service de Police de la Ville de Montréal*) for 2016 and 2017. For individuals involved in several IPV events (multiple victimization), only the earliest event for a given year was considered. Information on the gender and age of each victim as well as on the location of the assault was also provided. No other data on the events or individuals were available. Gender was measured binarily (male/female) while age was categorized into four groups (20–34, 35–49, 50–64, and 65 or over). Events were geolocalized by the SPVM at the dissemination-area (DA) level. DAs are spatial units defined by Statistics Canada with an average population of 606.7 inhabitants (std. dev. = 300.3 inhabitants) on the SPVM’s territory. DA population sizes obtained from the Canada Census^31^ were used as denominators for modelling the odds of IPV at the DA level.

Neighbourhood sociodemographic features were measured using DA-level data from the Canada Census.^31^ Neighbourhoods’ SES was operationalized using median DA income. DA-level proportions of single-parent households and residents living in the neighbourhood for less than five years were used for measuring single-parenthood and residential instability, respectively. Ethnocultural heterogeneity was measured by applying Shannon’s diversity index,^32^ using languages spoken at home as a marker of ethnocultural backgrounds. Languages were grouped into 16 separate categories inspired by a classification system based on world values and beliefs.^33^ Shannon’s index allows for measuring ethnocultural heterogeneity (*H*) at the DA level as follows:

$$H=\;-\overset{S}{\underset{i=1}{\sum }}(P_{i})\ln(P_{i})$$

where *P_i_* is the proportion of residents in language category *i* and *S* is the number of language categories represented in a given DA. Ranging from 0 to infinity, the index describes the uncertainty in predicting the language category of a randomly selected resident in a given DA. The index’s value increases following an increase in both the number of language categories (richness) and the evenness of the distribution of residents across language categories. All neighbourhood-level variables (median income, % of single-parent households, residential instability and ethnocultural heterogeneity) were categorized in population-weighted quintiles.

Some victims from the SPVM database had to be excluded due to missing data (unknown location, gender and/or age group) or if the event was located in a DA where no population was reported by the Canada Census. The final database used in this study comprises 8070 victims (representing 94.5% of all reported victims aged 20 years or older) distributed across 2 479 DAs on the SPVM territory.

### Statistical analysis

Multilevel logistic regressions were used for modelling the log-odds of IPV.^34^ Individual-level (Level 1) factors were gender and age group. Neighbourhood-level (Level 2) factors were median DA income, proportion of single-parent households, residential instability and ethnocultural heterogeneity. First, a null model and a model containing only individual-level factors were estimated. Then, associations between IPV and neighbourhood-level factors were estimated in four separate models, one for each neighbourhood-level factor adjusting for gender and age at the individual level. Finally, associations between IPV and neighbourhood-level factors were estimated from a single model including all four neighbourhood-level factors and individual-level factors. These estimations were obtained using the *glimmix* procedure in SAS (version 9.4).

## Results

The 9449 IPV events reported to the SPVM in 2016 and 2017 resulted in 8070 victims. Eight out of ten victims (78.48%) are women, and women show a crude victimization rate 3.3 times higher than that of men (3.97‰ vs 1.18‰) (Table 1). The youngest group (20–34 years old) is overrepresented among the victims (54.82%) and shows the highest crude victimization rate (4.98‰). More than half of the victims reported an IPV event in a neighbourhood of low SES (24.42% and 29.23% for the fifth and fourth quintiles, respectively) or in a neighbourhood with a high level of single-parent households (21.26% and 31.82% for the fifth and fourth quintiles, respectively). About half of the victims reported an IPV in a neighbourhood with high residential instability (23.22% and 25.07% for the fifth and fourth quintiles, respectively). In contrast, the distribution of victims is almost uniform across quintiles of ethnocultural heterogeneity.

**Table 1.** Distribution of IPV victims across individual and neighbourhood factors.

	Victims		
	Frequency	Proportion of victims (%)	Proportion in the population 20 years of age or older (‰)
Total	8070	100.00	2.63
Gender			
Female	6333	78.48	3.97
Male	1737	21.52	1.18
Age groups (years)			
20–34	4424	54.82	4.98
35–49	2685	33.27	3.34
50–64	810	10.04	1.10
65 or over	151	1.87	0.23
Median income (quintiles)			
Very high	695	8.61	1.18
High	1406	17.42	2.28
Medium	1639	20.31	2.68
Low	1971	24.42	3.19
Very low	2359	29.23	3.69
Single parenthood (quintiles)			
Very low	1042	12.91	1.67
Low	1239	15.35	1.98
Medium	1505	18.65	2.44
High	1716	21.26	2.80
Very high	2568	31.82	4.33
Residential instability (quintiles)			
Very low	1024	12.69	1.71
Low	1511	18.72	2.51
Medium	1638	20.30	2.68
High	1874	23.22	3.02
Very high	2023	25.07	3.15
Ethnocultural heterogeneity (quintiles)			
Very low	1627	20.16	2.55
Low	1643	20.36	2.52
Medium	1614	20.00	2.7
High	1628	20.17	2.71
Very high	1558	19.31	2.65

The odds of IPV victimization are higher for women than for men (OR = 3.61, 95%CI: 3.42–3.81, *p* < 0.001) and decrease with age (Model 1, Table 2). Median DA income is inversely associated with IPV victimization, with a progressive increase in the likelihood of IPV following a decrease in median income. The larger odds ratio (OR) was observed for the lowest income level (OR = 2.80, 95%CI: 2.47–3.17, *p* < 0.001; Model 2, Table 2). An increase of the likelihood of IPV are also observed across levels of single parenthood (Model 3, Table 2). Moreover, IPV victimization is associated with residential instability, but this association is not linear, as no gradient is observed across the levels of residential instability. Instead, in comparison to DAs with the highest level of residential instability, DAs from all other quintiles show a 50% increase in the odds of IPV (ORs ≅ 1.5) (Model 4, Table 2). Variation in the likelihood of IPV across levels of ethnocultural diversity does not follow a gradual trend. In contrast to DAs with very low ethnocultural diversity, IPV victimization is more likely in DAs with an average or high level of ethnocultural diversity (respectively: OR = 1.13, 95%CI = 1.01–1.27, *p* = 0.037; OR = 1.14, 95%CI = 1.01–1.28, *p* = 0.032). However, no significant differences were observed for neighbourhoods with a low or very high level of ethnocultural diversity (Model 5, Table 2).

**Table 2.** Associations between sociodemographic characteristics and IPV victimization.

	Model 1		Model 2		Model 3		Model 4		Model 5		Model 6	
	OR (95% CI)	Wald test (*p*-value)	OR (95% CI)	Wald test (*p*-value)	OR (95% CI)	Wald test (*p*-value)	OR (95% CI)	Wald test (*p*-value)	OR (95% CI)	Wald test (*p*-value)	OR (95% CI)	Wald test (*p*-value)
Fixed effects												
Level 1 variables												
Gender		<0.001		<0.001		<0.001		<0.001		<0.001		<0.001
Male	Reference		Reference		Reference		Reference		Reference		Reference	
Female	3.61 (3.42–3.81)		3.62 (3.43–3.82)		3.60 (3.41–3.79)		3.61 (3.42–3.81)		3.61 (3.42–3.80)		3.61 (3.42–3.80)	
Age groups (years)		<0.001		<0.001		<0.001		<0.001		<0.001		<0.001
20–34	Reference		Reference		Reference		Reference		Reference		Reference	
35–49	0.68 (0.65–0.71)		0.70 (0.66–0.73)		0.68 (0.65–0.71)		0.69 (0.65–0.72)		0.68 (0.65–0.71)		0.69 (0.66–0.72)	
50–64	0.22 (0.20–0.24)		0.23 (0.21–0.24)		0.22 (0.20–0.24)		0.22 (0.20–0.24)		0.22 (0.20–0.23)		0.22 (0.21–0.24)	
65 or over	0.04 (0.04–0.05)		0.04 (0.04–0.05)		0.04 (0.04–0.05)		0.04 (0.04–0.05)		0.04 (0.04–0.05)		0.04 (0.04–0.05)	
Level 2 variables												
Median income				<0.001								<0.001
Very high			Reference								Reference	
High			1.83 (1.61–2.08)								1.71 (1.47–1.97)	
Medium			2.07 (1.82–2.35)								1.85 (1.59–2.17)	
Low			2.41 (2.13–2.74)								2.06 (1.75–2.42)	
Very low			2.80 (2.47–3.17)								2.38 (2.01–2.81)	
Single parenthood						<0.001						<0.001
Very low					Reference						Reference	
Low					1.24 (1.09–1.40)						1.07 (0.94–1.22)	
Medium					1.52 (1.35–1.72)						1.20 (1.06–1.37)	
High					1.76 (1.56–1.98)						1.32 (1.15–1.50)	
Very high					2.67 (2.38–2.99)						1.88 (1.65–2.15)	
Residential instability								<0.001				0.005
Very low							Reference				Reference	
Low							1.51 (1.34–1.70)				0.96 (0.84–1.09)	
Medium							1.48 (1.31–1.67)				0.83 (0.73–0.95)	
High							1.55 (1.38–1.75)				0.84 (0.73–0.96)	
Very high							1.46 (1.29–1.65)				0.81 (0.70–0.93)	
Ethnocultural heterogeneity										0.046		0.038
Very low									Reference		Reference	
Low									0.99 (0.88–1.11)		1.03 (0.93–1.15)	
Medium									1.13 (1.01–1.27)		1.12 (1.01–1.26)	
High									1.14 (1.01–1.28)		1.04 (0.93–1.16)	
Very high									1.08 (0.96–1.22)		0.94 (0.84–1.05)	
Random effects												
Neighbourhood. β (SE)	0.59 (0.03)		0.52 (0.03)		0.49 (0.03)		0.58 (0.03)		0.59 (0.03)		0.46 (0.02)	
Intraclass correlation coefficient (ICC) (%)	15.28		13.64		12.99		15.01		15.23		12.29	

In a final model (Model 6, Table 2), the associations between IPV and neighbourhood variables were modelled jointly. In this fully adjusted model, median income is inversely associated with IPV, with ORs ranging from 1.71 (95%CI: 1.47–1.97, *p* < 0.001) to 2.38 (95%CI: 2.01–2.81, *p* < 0.001). Compared to the model controlling only for individual-level factors (Model 2, Table 2), the association between IPV and median income in the fully adjusted model is slightly lower. Similarly, the ORs for the single parenthood in the fully adjusted model are slightly lower than ORs controlling only for individual-level factors (Model 3, Table 2). Accounting for other neighbourhood-level variables drastically modified the estimated association between IPV and residential instability. Whereas the latter appeared to be a risk factor for IPV in the model accounting for only individual-level variables (Model 4, Table 2), the fully adjusted model suggests that residential instability could have a *protective* effect on IPV (Model 6, Table 2). Estimates for the association between ethnocultural diversity and IPV from the fully adjusted model do not suggest any gradual association; only individuals living in neighbourhoods with medium-level of ethnocultural diversity would have significantly higher odds of IPV than individuals from the least diverse neighbourhoods (Model 6, Table 2).

## Discussion

Studies of the relationship between sociodemographic characteristics of neighbourhoods and IPV have rarely been conducted outside the United States and have, to our knowledge, never been conducted in Montréal, Québec. Such analyses are essential for developing effective preventive strategies focused on reducing IPV. Our study aimed to analyse the association between neighbourhood features and IPV in the adult population in Montréal, Québec. Consistent with the SDT, our results suggest that several sociodemographic characteristics of neighbourhoods are associated with victimization occurring in the context of an intimate relationship.

SES is the factor most strongly associated with the risk of IPV victimization. The observed inverse association between neighbourhood-level SES and IPV is consistent with results of many other studies^13,20–23,25,27,28^ and highlights the extent to which SES contributes to the occurrence of IPV. Neighbourhood-level SES can influence many psychological processes. A low SES can be associated with a poor quality of life (e.g., disrepair, fear of others, lack of resources) which in turn generates more stress.^19^ Such conditions can increase levels of anxiety, depression, frustration and anger in individuals,^19,26^ which are known risk factors for perpetrated IPV.^2^ Social processes may also play a role in the association between neighbourhood-level SES and IPV victimization. Low SES is associated with weakened social ties in neighbourhoods, which may limit the ability of victims to seek help to avoid or stop violent behaviour.^9,13,19^ It may also lead to social isolation, limiting the extent to which values of intolerance towards violence are spread throughout the community.^9,19^

Results of this study suggest that a high proportion of single parenthood in neighbourhoods is associated with a higher likelihood of IPV victimization. However, this association seems to be confounded by other contextual variables, such as SES, which may be associated with exposure to several socio-environmental characteristics.^35^ When accounting for SES and other neighbourhood-level factors, the differences in risk of victimization observed in this study remain significant for most levels of single parenthood. This result is difficult to compare to other studies since neighbourhood-level single parenthood is seldom analysed independently. To our knowledge, only one other study isolated the effect of this characteristic and observed results similar to our results.^17^ In most research, single parenthood is included as part of a composite measure of disadvantage.^13,22^ However, there appears to be little collinearity between SES and single parenthood as suggested by the lack of important variation in standard errors (or in the size of the confidence interval) between estimates of these factors when modelled jointly (Table 2, Model 6) and estimates of these factors when modelled separately (Table 2, models 2 and 3). Thus, a composite index that integrates these two factors could have different spatial variations than those observed with SES and single parenthood separately. The inclusion of single parenthood in a composite measure could therefore mask the independent effect of this characteristic on IPV as suggested in this study, or even produce estimates that reflect neither the effect of SES nor that of single parenthood.

The prevalence of single parenthood in neighbourhoods could be associated with IPV victimization through several mechanisms. A high proportion of single-parent households may limit formal social control since residents are less likely to be involved with neighbourhood institutions (e.g., community organizations) due to lack of availability.^18,36^ The proportion of single-parent households may also be related to the propensity of residents to be actively involved in their neighbourhoods. This in turn influences the development and maintenance of social ties among residents, which is the basis for informal social control (e.g., supervising youth, intervening to limit neighbourhood disturbances).^18,36^ Weaker informal social control may reduce a local community’s ability to deter potential perpetrators from acting out, while weak social ties limit the amount of support provided to victims.^13,19^

The effects of residential instability and ethnocultural diversity on IPV victimization are difficult to assess. The association of residential instability with IPV appears to be confounded by other neighbourhood characteristics. When accounting only for individual-level factors, the risk of victimization was lower in neighbourhoods with very low residential instability compared to other neighbourhoods. However, the fully adjusted model suggests that this feature may have a protective effect on IPV. Other studies have also reported an inverse association between residential instability and risk of IPV.^20,23^ One possible explanation is that, instead of being a marker of social ties, residential instability would rather be indicative of economic and racial discrimination as well as the inability of residents to move out of the neighbourhood.^37^ Such conditions could lead to isolation and frustration and ultimately affect mental health (e.g., by promoting anxiety or depression^38^), which in turn can influence the risk of IPV.^2^

The effect of ethnocultural heterogeneity on IPV is difficult to characterize since no clear trends emerged from our results. This result is in line with other studies: few studies observed an association between ethnocultural heterogeneity and IPV in accordance with the SDT.^13,22,24,25,30^ According to this theory, ethnocultural heterogeneity is positively associated with IPV as it contributes to reduced communication among residents and thus limits social cohesion and the effectiveness of informal social control; it is assumed that cultural differences generate fear and lack of trust.^12^ However, the effect of ethnocultural heterogeneity on social cohesion is not well established in the literature and some work suggests that it may even be beneficial for some communities.^15,16^ In disadvantaged neighbourhoods, strong ethnocultural heterogeneity may be associated with higher levels of social cohesion,^16^ a potential protective factor for IPV.^13^ The role of this characteristic should therefore be clarified in order to better understand its potential effect on IPV and the mechanisms through which it acts. Furthermore, it is important to note that most studies referring to ethnocultural diversity measure the proportion of immigrants and not diversity *per se*. The proportion of immigrants may not adequately capture geographic variations in ethnocultural diversity at the local level. On the one hand, neighbourhoods with comparable proportions of immigrants may have different levels of ethnocultural diversity. The composition of some neighbourhoods may be dominated by a single immigrant community, while other neighbourhoods may be composed of a wide variety of ethnocultural communities. On the other hand, the proportion of immigrants may underestimate ethnocultural diversity in areas where a significant proportion of the population were born in Canada but where the ethnocultural backgrounds of families vary (e.g., second or third generation immigrants).

This study is not without limits. The use of police data provides a complete population coverage (vs sampling) and greater statistical power than survey data. However, they do not identify all victims of IPV and may underestimate the extent of the phenomenon. Indeed, the General Social Survey suggests that a significant proportion of IPV victims (7 out of 10 victims) do not report incidents to police authorities.^7^ Police data therefore underestimate the number of IPV victims. Conversely, the measures used in surveys generally allow for retrospective analysis (e.g., questioning about victimization over the past 5 years), which is likely to lead to a larger number of victims. In addition, while survey data consider different forms of IPV, police data is likely to be biased towards the most severe cases of IPV^39^ as victims would tend not to report incidents deemed less severe, namely psychological violence. However, if the propensity to report an IPV incident to the police or the severity of IPV events varies according to neighbourhood type, police data could provide a biased portrait of the spatial variation of IPV victimization. The use of comprehensive data could have led to weaker associations between neighbourhood characteristics and IPV than those reported in this study. These potential biases are difficult to assess as we are unaware of studies reporting on such biases.

The police database only contained information on victims by age and gender and it was therefore not possible to account for the effect of other individual-level factors, namely individual-level SES, which is a potential confounding factor.^17,20,23,29^ Because the contextual measures used here are derived from the aggregation of individual data, the estimated effect of neighbourhood-level factors is likely to be attributable, at least in part, to the geographical variation in individual characteristics. It is therefore possible that the observed associations would have been of a lesser magnitude had the models been adjusted for additional individual-level factors.

## Conclusion

This study observed a significant variation in IPV victimization at the neighbourhood level. The prevalence of IPV was found to be higher in neighbourhoods with low SES and high proportions of single-parent households. Prevention strategies could target these high-risk settings, where IPV is more prevalent, by improving living conditions in neighbourhoods. Indeed, exposure to a disadvantaged environment can increase the risk of IPV by acting through several mechanisms. Collective efficacy (i.e., ability of community to act for a common purpose such as preventing and controlling neighbourhood violence) is a key concept in SDT.^13,14^ According to this theory, a lack of collective efficacy in disadvantaged neighbourhoods can contribute to a higher risk of IPV.^13^ Neighbourhood disorder is also often integrated in the SDT, and refers to the social (e.g., crime) and physical (e.g., graffiti) characteristics that could be sign of a poor social control within the community.^40^ Social and physical disorder could be more prevalent in disadvantaged neighbourhoods and have been identified as a risk factor for IPV.^26,28^ Finally, studies suggest that living in disadvantaged neighbourhoods can foster stress and exposure to social norms that tolerate violence, which can increase the prevalence of IPV.^10,19^ Preventive strategies targeting these social and physical conditions of neighbourhoods could be implemented to reduce the prevalence of IPV. Community-based programs aimed at increasing social cohesion, residents’ social participation, the capacity for collective action, bystanders’ intervention as well as improving physical environment may be effective in combating IPV.^9,41^ This population-based approach has many advantages as it can benefit a large number of individuals at relatively low cost.^41^ The greatest benefit could be observed in the most disadvantaged neighbourhoods, where social ties and collective efficacy are often more limited.^13^

Future work on the processes underlying the connections between neighbourhood sociodemographic characteristics and IPV is required in order to better identify the specific factors that should be the focus of local interventions. As suggested above, collective efficacy, neighbourhood disorder, and social norms could mediate the relationship between neighbourhood sociodemographic characteristics and IPV, but empirical studies analysing these potential pathways are still scarce. Moreover, further knowledge of the structuring effect of neighbourhood-level SES on local social factors is needed. For example, neighbourhood SES may influence residential instability and ethnocultural diversity, which in turn limits the creation of social ties within the community, a potential risk factor for IPV.^13^ Theoretical frameworks in health geography also assign such a role to SES,^42^ but few studies have actually investigated the structuring effect of SES on the social environment of neighbourhoods. Multilevel mediation analyses, which simultaneously consider several individual and contextual factors, are required to assess such an effect; however, data for this type of analysis is currently scarce.
